# The impact of cystic fibrosis transmembrane conductance regulator (CFTR) modulators on the pulmonary microbiota

**DOI:** 10.1099/mic.0.001553

**Published:** 2025-04-09

**Authors:** Joshua K. Robertson, Joanna B. Goldberg

**Affiliations:** 1 Department of Biology, Emory University, Atlanta, Georgia, USA; 2 Department of Pediatrics, Emory University School of Medicine, Atlanta, Georgia, USA; 3 Emory+Childrens Center for Cystic Fibrosis and Airway Disease Research, Emory University School of Medicine, Atlanta, Georgia, USA

**Keywords:** cystic fibrosis, cystic fibrosis transmembrane conductance regulator (CFTR) modulators, elexacaftor, tezacaftor and ivacaftor (ETI), highly effective modulator therapy, microbiology, respiratory infections, sputum cultures, Trikafta

## Abstract

Cystic fibrosis transmembrane conductance regulator (CFTR) modulator therapy has significantly changed the course of the disease in people with cystic fibrosis (CF) (pwCF). The approved triple therapy of elexacaftor, tezacaftor and ivacaftor (ETI), commercially known as Trikafta, increases CFTR channel function, leading to improvements in sweat chloride concentration, exercise capacity, body mass index, lung function and chronic respiratory symptoms. Because of this, the majority of pwCF are living longer and having fewer CF exacerbations. However, colonization with the common CF respiratory pathogens persists and remains a major cause of morbidity and mortality. Here, we review the current literature on the effect of ETI on the respiratory microbiota and discuss the challenges in addressing CF lung infections in the era of these new life-extending therapies.

## Introduction

Cystic fibrosis (CF) is an autosomal recessive disorder characterized by pancreatic insufficiency, recurring respiratory infections and other comorbidities [1]. The disease is caused by mutations in the cystic fibrosis transmembrane conductance regulator (CFTR), a chloride channel present in epithelial and non-epithelial cells that helps to maintain ion and water balance [2,3]. Mutations in CFTR have been grouped into classes, with class II mutations being the most common and affecting 88% of people with CF (pwCF); F508del, a class II mutation, is the most common mutation overall in CFTR. Class II mutations cause CFTR misfolding and inhibition of trafficking to the membrane. Other classes of mutations can reduce synthesis, stop the ion channel from opening or stop the protein from being synthesized at all [4]. These disruptions lead to dehydration of the cell surface, mucociliary defence inhibition and inability to buffer gastric acidity [2], which facilitates low weight gain, male infertility and chronic bacterial respiratory infections with associated deteriorations in lung function [2]. Fortunately, CFTR modulator therapies have revolutionized the treatment of CF and allowed healthcare providers to transition from managing infections and symptoms to treating the underlying condition.

**Fig. 1.**
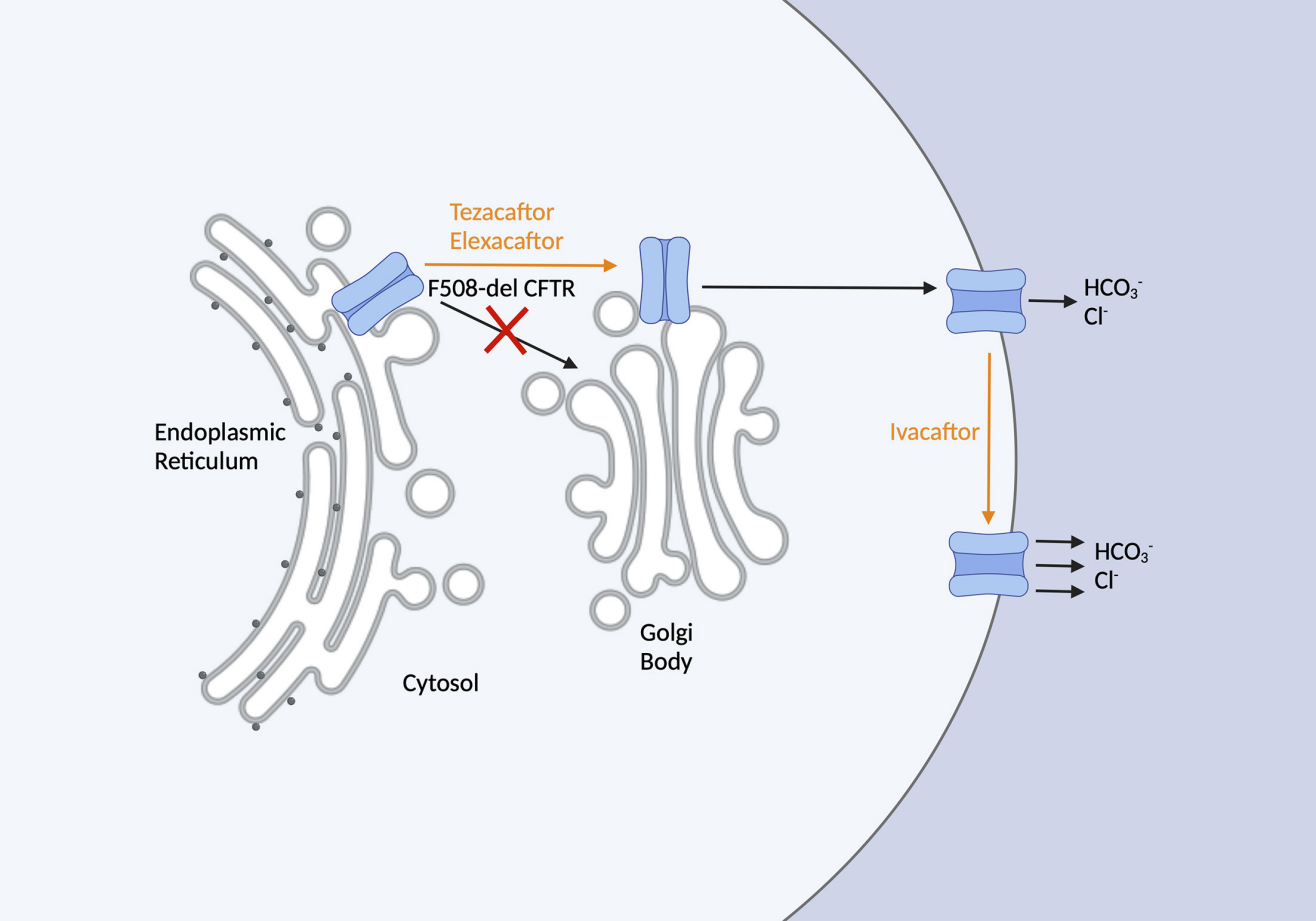
Sites of inhibition by Trikafta (ETI). Adapted from Zaher *et al.* [4]. TEZ and EXA are two CFTR correctors that increase the amount of CFTR protein that reaches the cell membrane by acting on protein trafficking and modification. IVA is a potentiator that acts on gating mutations and improves the opening of the CFTR protein and thereby increases ion transport.

CFTR modulators are compounds that bind to the CFTR protein and improve its function, which returns water and ion balance to standard levels. There are two types of CFTR modulators: potentiators and correctors [4]. Ivacaftor (IVA; Kalydeco) is currently the only FDA-approved potentiator; in 2012, it was approved for pwCF with a mutation in G551D or in one of the 96 other gating mutations. IVA improves the opening of the CFTR protein and thereby increases ion transport. Elexacaftor (EXA) and tezacaftor (TEZ) are two CFTR correctors that increase the amount of CFTR protein that reaches the cell membrane by acting on protein trafficking and modification (Fig. 1). The combination of these three therapies was demonstrated to be effective in treating the most common CFTR mutation, F508del, so in 2019, the FDA approved the triple therapy of elexacaftor, tezacaftor and ivacaftor (ETI), referred to as highly effective modulator therapy and commercially known as Trikafta in the USA [4] for pwCF with at least one F508del allele. The European Medicines Agency granted marketing authorization for ETI in the European Union and the UK in 2020, where the drug is known as Kaftrio [5]. With the F508del allele being targeted, over 80% of pwCF are considered eligible for ETI treatment. Interestingly, ETI has also been observed to be clinically efficacious for pwCF with certain non-F508del mutations [6].

ETI therapy has been unprecedented in improving the lives of pwCF. Whilst life expectancy for pwCF had increased from 4 to 5 years in 1954 to ~48.4 years in 2019, the implementation of Trikafta therapy has begun to increase the life expectancy even further [7,9]. Studies have demonstrated improvements in sweat chloride concentration, exercise capacity, body mass index, bone mineral density, lung function and chronic respiratory symptoms [10,12]. Cost remains as a significant barrier to distributing these life-saving therapies, but recent developments, like the National Institute for Healthcare Excellence in the UK finding ETI therapy to be cost-effective and approving it for National Health Service coverage, provide an optimistic outlook for the future [13].

Respiratory disease remains one of, if not the most, significant comorbidities leading to lung transplant, decreased quality of life and loss of life in pwCF [2,8]. The lungs of younger pwCF are typically dominated by *Staphylococcus aureus* and a smaller proportion of other pathogenic bacteria. However, as pwCF age, the pulmonary composition moves increasingly towards *Pseudomonas aeruginosa* and other pathogens like *Mycobacteria* species, *Stenotrophomonas maltophilia* and the *Burkholderia cepacia* complex, which are associated with increased morbidity and mortality [2,8, 9, 14]. With this in mind, it is clear that the makeup of the pulmonary microbiota has a significant impact on clinical outcomes. Trikafta therapy has been demonstrated to significantly change the composition of the pulmonary microbiota [12]. The anticipated changes in the lung microbiota could be incredibly complex to predict given the existing diversity of infections in the CF population. For instance, initiating Trikafta in a middle-aged pwCF with a chronic *P. aeruginosa* infection could lead to a significantly different bacterial respiratory microbiota than starting the therapy with a paediatric pwCF with mainly *S. aureus* present. One of the challenges to studying this relatively rare disease is the small pwCF cohort, which makes clinical predictions difficult. Furthermore, an unintended consequence of the effectiveness of Trikafta is that it results in decreased sputum production, the analysis of which has generally been used as the gold standard by the clinical microbiology laboratory to identify, enumerate and characterize respiratory pathogens responsible for chronic infection and subsequently design appropriate treatments. Assessing the respiratory microbiota composition following modulator therapy requires comprehensive longitudinal clinical studies, retrospective analysis and more basic science research to better understand the nature and adaptation of the pathogen population to the lung and therefore to better protect pwCF.

In this review, we will look at recent publications relevant to the changing pulmonary microbiota after CFTR modulator therapy (focusing mainly on Trikafta) in pwCF. Assessments of changes in diversity by existing studies will be evaluated. Changes in small molecule composition and the immune response will also be discussed to identify potential causes and factors contributing to the shifts in microbial populations. We will also summarize studies looking at the impact of CFTR modulators on the most common pathogens in CF respiratory disease, *P. aeruginosa* and *S. aureus*, along with briefer overviews of the less common microbes based on standard microbiological analysis. Lastly, we will present a brief survey of changes observed in microbiota other than that of the respiratory tract in pwCF after modulator therapy, which could provide additional insight into the changes observed in the pulmonary system.

## Microbiota diversity after CFTR modulators

With Trikafta therapy being approved in 2019 [4], multiple studies have assessed the diversity of the pulmonary microbiota before and after treatment for different cohorts on different time scales; summaries of these studies have been published [15,19], but these reviews were unable to include the more recently published studies with Trikafta and following pwCF for longer periods of time, which is important when evaluating this recently approved therapy.

Since then, there are several newer studies that have looked at changes in the diversity of the pulmonary microbiota after initiating Trikafta treatment. Most studies used 16S rRNA sequencing to identify the bacteria and gather raw data. Several different measures of diversity (Shannon index, Simpson diversity and Bray–Curtis dissimilarity) were used. Definitions of these diversity measures and other terms used in the review are provided in Table 1. A variety of statistical methods including the data mining t-test and the Wilcoxon signed-rank test were employed in the studies that will be discussed.

**Table 1.** Definitions of biodiversity terms used in this review

Biodiversity term	Definition
Alpha diversity	It is the comparison of the species composition within a community [26].
Beta diversity	It is the comparison of the species composition between communities [26].
Bray–Curtis dissimilarity	Measures beta diversity. It is a measure of dissimilarity between two communities calculated with species relative abundances. This statistic is widely used and known to be robust [26].
Evenness	It is how consistent the abundance of all species is within a community [26].
Euclidean distance	Measures beta diversity. This less-used dissimilarity measure is calculated by determining the distance between two points on principal coordinate axes, which is the same as the dissimilarity across those variables. It uses absolute abundance, which can be more representative of the diversity in some communities [61].
Richness	It is the number of species in a community [26].
Simpson index	Measures alpha diversity. This index highly weighs evenness. It calculates the probability of two randomly sampled individuals being the same species. The inverse of this index is widely used [26].
Shannon index	Measures alpha diversity. This popular index uses estimates of species’ proportional abundance and highly weighs richness. It assumes that every species in the community is sampled, the sample is random and the sample was collected from an infinitely numbered community. Some weaknesses of the measure are susceptibility to error when not all species are sampled and difficulty finding statistical differences [26].
Pielou’s evenness	Measure of evenness using the Shannon index [26].
ΔUniFrac	Measures beta diversity. Uses rRNA data to measure phylogenetic distances between microbial communities [62].

Table 2 summarizes the design, methodology and results of recent microbiota studies. In the following paragraphs, we will summarize the variability in key areas, highlight the important findings from these studies and discuss the significance to our understanding of the changes occurring in the CF lung. Sample sizes were a limiting factor in the majority of the studies. Nichols *et al*., in the PROMISE-micro prospective study, had the largest sample size of 236 [12], whilst Schaupp *et al*. had 79 participants [20]. The other four microbiota studies had less than 25 participants. The lower participant studies still provide valuable data and insight; however, the reduced statistical power and increased probability of sampling errors can diminish reliability and make it more difficult to reach a consensus. Enrolling more participants in CF post-ETI studies is a challenge that requires immense coordination and effort from scientists, public health experts and advocates. With CF and associated infections impacting so many lives and having such a significant financial cost, it remains essential for cost-effective and highly powered studies to be conducted.

**Table 2.** Summary of studies evaluating pulmonary microbiota diversity changes post-Trikafta therapy

Study	Sample size	Age group	Timeline	Sample type	Sequencing method	Diversity measures	Key findings
[23]	7	≥18 years	Weekly (18–40 samples per subject)	Sputum (home-collected)	16S rRNA	Shannon and ΔUniFrac (beta)	Significant increase in Shannon (alpha) and ΔUniFrac (beta); linear diversity increase in 5 out of 7 subjects over time.
[12]	236	≥12 years	1, 3 and 6 months (part of a 2-year study)	Sputum	16S rRNA	Shannon, Simpson and Bray–Curtis (beta)	Alpha diversity (Shannon and Simpson) increased at 1 and 6 months; Bray–Curtis showed beta diversity shifts before and after ETI; pathogen removal negated changes.
[24]	24	≥12 years	14 and 50 weeks	Deep cough swabs and induced sputum	Metagenomic shotgun	Shannon, Simpson, Pielou’s evenness and Euclidean distance	Bacterial load decreased; Pielou’s evenness increased in swabs; no change in Shannon/Simpson; variability by sputum production; no difference in composition of community before and after ETI.
[20]	79	≥12 years	1, 3 and 12 months	Sputum	16S rRNA	Shannon, Pielou’s evenness and richness	Significant increase in Shannon and Pielou’s evenness at all time points; richness up at 3 and 12 months; bacterial load unchanged.
[22]	24	≥18 years	202 days+/−108 sd	Sputum	16S rRNA	Shannon, Pielou’s evenness, Bray–Curtis	Significant increase in Shannon and Pielou’s evenness; beta diversity shifted, but individual samples remained similar pre/post.
[25]	20	12–20 years	Pre-ETI, biweekly for 12 weeks	Oropharyngeal swabs	Metagenomic shotgun	Shannon, Simpson, evenness, and Bray–Curtis	Slight Shannon and Simpson diversity increase; no evenness change; community changed (beta diversity) after 2 weeks; commensal bacteria increased; *S. aureus* reduced.

The age groups of the cohorts were another significant limitation. Several of the studies included participants 12 years of age and older, but some only had adults, whilst no individuals younger than 12 were included in any of the studies. With Trikafta only being approved for ages 2–5 in the USA in 2023 [21], these limitations are understandable but still leave an important knowledge gap. Since the composition of the CF pulmonary microbiota changes with age [2,8, 9], the effect of ETI on paediatric patients has the potential to be unique and require specific attention. None of the studies discussed have timelines that last over a year. Only PROMISE-micro is part of a larger prospective study over 2 years, but more and continued longitudinal studies would be helpful for gaining a better understanding of the short- and long-term changes occurring in the microbial composition of the lung.

Most of the following studies, despite differences in timelines, methods and analysis, found increases in alpha diversity and beta diversity. Each still has unique key findings and limitations that should be discussed. Sosinski *et al*. [22] had significant limitations of sampling not regularly taking place and the study being conducted only a few years after Trikafta approval. Sampling occurred at the most recent visit prior to starting Trikafta and the most recent visit where sputum was collected. They did not find a significant increase in amplicon sequence variant between their samples, but they did find a significant increase in Pielou’s evenness as well as in Shannon diversity. Analysis of beta diversity between and across subjects showed that there was a significant change in the microbiome after Trikafta, but samples of an individual before and after treatment were still more similar to each other than samples across individuals [22].

A study by Schaupp *et al*. [20] identified an increase in Shannon diversity at 1, 3 and 12 months. Pielou’s evenness also increased at all these time points, whilst the richness of ribosomal sequence variants was found to increase at 3 and 12 months. Importantly, total bacterial load stayed the same in the participants throughout the entire study [20], which represents an important finding.

Martin *et al*. [23] instructed participants to collect sputum samples at home weekly and evaluated the change in both alpha and beta diversity. The pwCF were on Trikafta for different periods of time, and sample collection was at the participants’ homes and at their discretion. These factors limited the continuity and reliability of the samples collected, but 18–40 sputum samples were still collected for each person on triple therapy. The study found a significant increase in alpha and beta diversity per day per subject, with Shannon diversity and ΔUniFrac being used to measure diversity, respectively. A random forest regression analysis found a linear association between microbiome diversity and time since starting Trikafta in 5 out of 7 participants, which supports that composition was constantly diversifying in these subjects [23].

PROMISE-micro, the large prospective study previously discussed, found an increase in alpha diversity in their cohort. Interestingly, when CF pathogens were computationally removed from the diversity calculations, there was no significant change in alpha diversity, which suggests that the change in diversity is being mediated by a decrease in pathogen abundance [12]. Beta diversity using the Bray–Curtis index also had significant differences between pre- and post-Trikafta compositions. A similar computational removal of pathogens also removed the significant difference in beta diversity, which gives even more evidence that diversity changes are pathogen-mediated [12]. With this study only having evaluated changes for 2 years after the introduction of Trikafta to the market, how its findings related to diversity are relevant to changes in the longer term is somewhat uncertain. However, the high number of participants, more consistent time frames and large quantities of data make this study strong evidence for ETI therapy increasing pulmonary microbiota diversity.

Pallenberg *et al*. [24] collected deep cough swabs and/or induced sputum in a cohort of pancreatic-insufficient pwCF. The unique population of the cohort could produce a different result than a more representative sample of pwCF, which should be considered when evaluating the data. In this cohort, the total bacterial load decreased [24], which contradicts the results from Schaupp *et al*. where it stayed the same [20]. Future pulmonary microbiota studies should continue to evaluate bacterial load, as it could have a significant impact on disease and quality of life in the future. It was also found that there were overall trends with respect to the ability to produce sputum and the presence of CF pathogens. Those patients who could not produce sputum but could only provide deep cough swabs had post-ETI species more similar to that of pre-ETI, suggesting a discordance between these different patients (or these sampling sites). The patients who could only be examined by deep cough swabs showed a higher proportion of rare species after ETI. When only comparing deep cough swab samples, a decline in bacterial load and an increase in Pielou’s evenness were observed in pwCF after ETI, but Shannon and Simpson diversities were not different. It was found that the microbial composition of the same pwCF before and after treatment had no significant difference. Samples collected before treatment had more internal variation, which the authors say could suggest that the microbial samples are becoming more similar after ETI. Importantly, the authors also found that individual patient responses to treatment could not necessarily be predicted and may depend on age and/or prior infection status [24].

Steinberg *et al*. [25] utilized oropharyngeal swabs in a younger cohort taken by the parents after training. They observed a small increase in Shannon and Simpson diversities after the initiation of ETI; however, the evenness of the community did not change. The microbial community changed within the first 2 weeks, and this was found to be due to an increase in the abundance of commensal bacteria. It is noteworthy that in this patient cohort, *S. aureus* was the pathogen most identified at the initiation of this study (17 out of 20), compared to only 2 out of 20 for either *P. aeruginosa* or *Haemophilus influenzae*. This did decrease during the 12 weeks following ETI initiation (13 out of 20) compared to *P. aeruginosa,* which decreased to 1 out of 20, and *H. influenzae* was not detected. This observation confirms what has generally been seen in sputum samples from younger pwCF, that *S. aureus* is one of the most prominent bacterial pathogens [9]. Importantly, ETI was effective in increasing the percent predicted forced expiratory volume (ppFEV1) significantly during the 12-week study.

Studies on changes in the diversity of the pulmonary microbiota after Trikafta therapy largely support increased alpha and beta diversity after starting treatment. It is uncertain when or if these changes will stabilize, but the current findings are promising. They suggest that the increase in bacterial diversity is mediated by a reduction in pathogenic bacteria [12,25] or an increase in commensal bacteria [25], potentially providing a hopeful clinical outlook for the future. Additional longitudinal studies are needed to better characterize how these changes in diversity trend in the long term and what outside factors contribute to these changes.

These studies use different measures of diversity. Many used the Shannon diversity index, which has been criticized for biases and inaccuracies [26,28], so more robust measures of diversity should be considered, especially with small sample sizes. However, the ubiquity of finding increasing diversity across the various time frames in all these studies supports that the findings are not due to biassed measures. Cooperation and optimizing these details will be essential to studying changes in diversity going forward.

## Changes in the small molecule environment

By returning function to the CFTR protein, Trikafta dramatically alters the physiology in pwCF. Decreases in sweat chloride [11,12] and ameliorated viscoelastic properties of sputum [20] are direct results of the restored function, but how aa, proteases, lipids and other molecule concentrations change is less obvious. Studying these changes could allow us to better understand the causes of shifts in the microbiota. Metabolites were quantified through MS in all the following studies [20,22, 23, 29].

Some of the studies that looked at shifting diversity in the pulmonary microbiota also looked at changes in the metabolome and small molecule environment. Sosinski *et al*. examined the metabolome and noted a significant decrease in molecular features and some metabolites. They found that there was a large amount of beta diversity variation in metabolites in samples collected from pwCF after ETI, suggesting that the composition of sputum is more variable across individuals; the composition was more similar between patients prior to the onset of CFTR modulator therapy [22].

The prevalence of individual molecules before and after Trikafta therapy has also been examined. Upper respiratory mucosal concentrations of micronutrients, copper, manganese and zinc were decreased in patients on Trikafta [29]. A grouping of metabolites in another study revealed that the most significant decrease was in aa and small peptides. This decrease was mostly facilitated by dipeptides and tryptophan [22]. Contrastingly, with a small panel of pwCF, another study found the metabolome to consistently change, but there were no metabolites that consistently changed with time across individuals [23]. Still, another group found that the protein composition of the sputum moved closer to that of healthy controls up to 12 months after starting Trikafta [20]. Elevated levels of tryptophan and other aa are associated with the chronic CF lung [30], so the observation that these values were lower after the start of ETI therapy would support the changes observed in the microbiota. Some potential mechanisms of these changes could be microbes adapted to the chronic environment being less fit and improved immune function.

Although some studies show an association between changes in both the diversity of the microbiome and that of the metabolome [23], the consensus about the changes in metabolome diversity remains much less clear than the microbiota. The causation is an especially important yet unclear factor. We have mostly discussed how the changing small molecule environment could lead to changes in the microbiota, but it is also plausible that a change in microbe composition and an associated decrease in protease production and metabolism could mostly mediate the changes in the small molecule environment. Both hypotheses are likely true to some degree, but a better mechanistic understanding could open the way for future interventions and therapies to improve CF lung function and other chronic infections. Either way, the levels of micronutrients, ions and peptides have important implications for bacterial growth and the health of pwCF, so more research is needed to create a greater consensus on Trikafta’s effects on the small molecule environment in the lung.

## Changes in the inflammatory response

Another significant factor that contributes to microbiota composition is the immune response from the host. A reciprocal relationship exists between the small molecule environment and the immune system, whereby modulation of one impacts the other. As pointed out in a review by Caverly *et al*., the effect of modulator therapies on inflammation and the changes in the inflammatory response following treatment are very complex and difficult to study [14]. In the context of the pulmonary microbiota, these differences in the inflammatory response could have very large effects on the composition. Typically, high levels of neutrophils, cytokines, proteases and other inflammatory factors are present in the CF lung, causing lung damage and worse morbidity. Studies of the inflammatory response after starting Trikafta have shown a decrease in the response across several measures. Several studies found that cytokine concentrations were lower after ETI therapy compared to before [20,29, 31]. In one study, IL-1β, a cytokine associated with lung disease, had its concentration decrease after 1 month and stayed lowered for the 12 months that the study continued. IL-6 and IL-8, other lung disease inflammation markers, were more variable and likely need a study with larger sample sizes to better characterize the changes [20]. Another study looking at iron regulation in chronic CF macrophages found that dysregulated iron metabolism in macrophages led to higher environmental iron. With Trikafta treatment, macrophage expression became more similar to healthy controls and environmental iron decreased [32], showing not only the positive impact of ETI on the immune response but also the close relationship with the immune response and the small molecule environment. Blood immune cell concentration, including neutrophils, appears to be significantly decreased after the start of Trikafta treatment, returning nearly to levels of healthy controls [31].

**Fig. 2.**
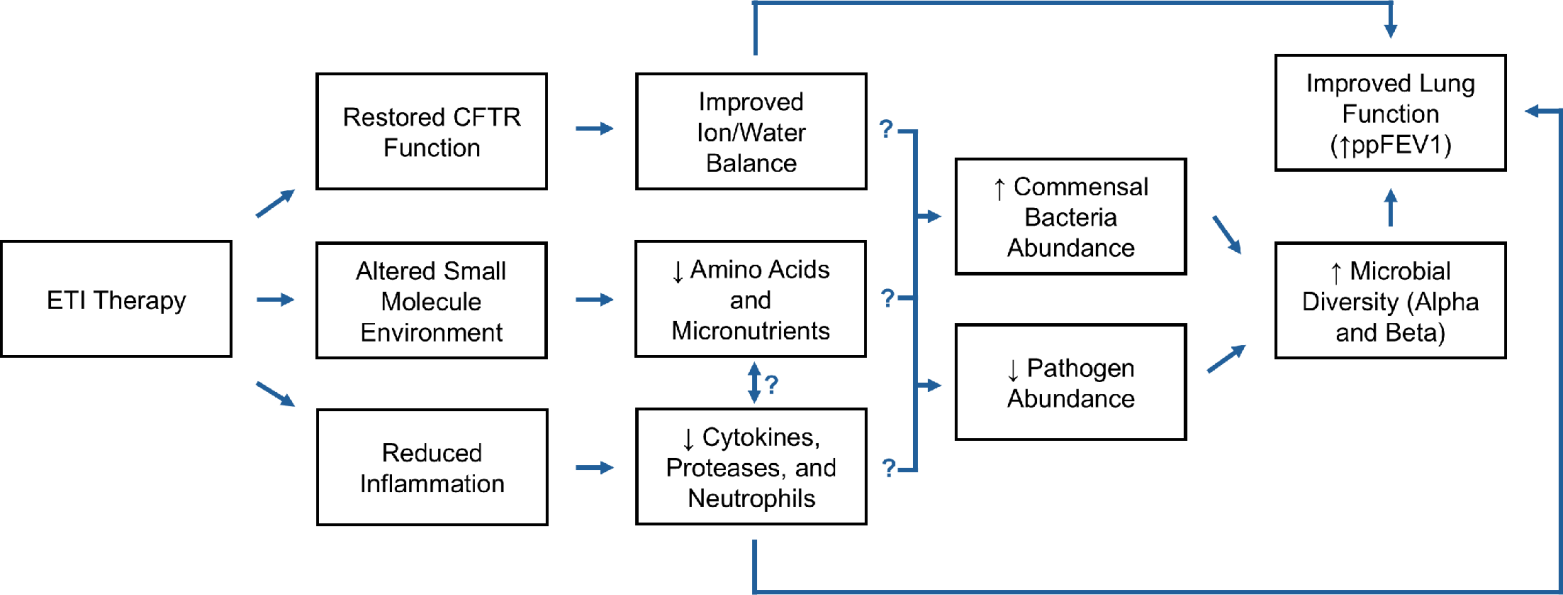
Proposed mechanism of Trikafta increasing microbial diversity and improving lung function. The down arrow indicates a decrease in abundance and the up arrow indicates an increase in abundance.

High protease activity is a significant source of lung damage in chronic infections. Schaupp *et al*. found that neutrophil elastase, cathepsin 3 and proteinase 3 levels lowered after starting ETI, but tumour necrosis factor alpha levels stayed the same [20]. The consensus of less inflammation after Trikafta is a promising indicator for the future of the CF lung environment. Less inflammation in the lung due to returned CFTR protein activity is a potent potential mechanism for an increase in microbiota diversity. Pathogens adapted to the inflammatory environment could lose relative abundance in the community as commensal bacteria that are more fit for the new environment emerge, but other potential mechanisms, like improved immune function directly inhibiting pathogens, are also supported by the data (Fig. 2). Finding a direct relationship between the changes in the immune system and microbial composition is a challenging task. More studies, especially those like Schaupp *et al*. [20], studying the microbiome, small molecule environment and immune response at the same time, are needed to find out which inflammatory factors stay the same or change and to associate these changes with changes in the small molecule environment and the microbiota.

## Effect of CFTR modulators on lung microbes

With the clear changes in the lung microbiota, metabolome and inflammatory response, each individual microbe in the CF lung is facing a significantly different environment that undeniably provides new evolutionary pressures, which have the potential to increase abundance, decrease abundance and promote evolution. Since the approval of ETI, several studies have assessed the presence or absence of specific CF pathogens using standard clinical microbiological analysis of samples from pwCF. An early case-controlled study on ETI was performed in Palermo, Italy. Migliorisi *et al*. [33] monitored two groups of 13 pwCF. One group had worse disease and, because of this, received compassionate use of ETI; none of the control groups were eligible for this therapy. Sputum culture analysis was analysed in both groups, with the major difference being the prominent pathogen identified. Before ETI, each member of the treated group (13 out of 13) had *P. aeruginosa* which decreased (7 out of 13) after treatment. Interestingly, *S. aureus* increased from 6 out of 13 to 8 out of 13 after treatment in these pwCF. All members of the control group harboured *S. aureus*, and 8 out of 13 also had *P. aeruginosa*. Perhaps not unexpectedly, the microbiology did not change during the observation period in this control group but suggests that *P. aeruginosa* was likely responsible for the worse lung disease in the treatment group, as has been previously reported [34].

Mianowski *et al*. [35] monitored 198 French adult pwCF 3 years prior and 1 year post-Trikafta. Importantly, they noted an increase in ppFEV1 and a decrease in the number of pulmonary exacerbations, the need for physiotherapy, the number of patients hospitalized and the time of hospital stay after ETI was initiated. As far as microbiology is concerned, they used sputum cultures to monitor bacterial colonization of seven pathogens of interest. They noted an overall decrease in bacterial culture density and a significant decrease in the number of patients colonized with *P. aeruginosa*, methicillin-sensitive * S. aureus* (MSSA), methicillin-resistant *S. aureus* (MRSA), *S. maltophilia*, *Achromobacter* and nontuberculous mycobacterium (NTM) species. There was no significant decrease in the number of patients with the *B. cepacia* complex or *H. influenzae* (but the number of these patients was relatively low). In looking at the mean bacterial density, when it could be analysed, most showed a decrease in the density in the sputum for most of the pathogens after treatment; however, the mean level of *H. influenzae* in the sputum was shown to increase after a year of ETI. These authors also noted a decrease in the number of pwCF with chronic *P. aeruginosa* infections after ETI and no new *P. aeruginosa* colonization. They speculated that the increase in *H. influenzae* may be an indirect indicator of the decrease in *P. aeruginosa*, as one product of *P. aeruginosa*, pyocyanin, has been shown to inhibit *H. influenzae* growth. This suggestion further validates the importance of monitoring the bacterial community in such analyses rather than focusing on one pathogen.

Beck *et al*. [36] performed a retrospective chart review of the bacterial culture results from the University of Iowa on pwCF (≥12 years old) before and after ETI (for at least 12 months). There were 124 subjects in this study. They found that cultures positive for *P. aeruginosa*, MSSA and MRSA were 54%, 33% and 31%, respectively, before Trikafta and decreased to 30%, 32% and 24% after ETI therapy was initiated. However, it was noted that sputum was the more prevalent source for culture prior to the initiation of ETI, whilst throat cultures were the main source post-ETI. Thus, care should be taken in interpreting these types of comparisons when using different types of samples.

An observational cohort study was carried out by Dittrich *et al*. on data from the German CF registry from January 2016 to December 2022 [37]. They also found improved lung function after modulators and reduced sputum availability, so this study also used culture-based analyses of throat samples or sputum. These authors detected *S. aureus* in 54% of all samples prior to ETI and then a rapid reduction in *S. aureus*-positive samples to 44%, 44% and 40% after 3, 12 and 21 months of ETI therapy, respectively. Similar results were seen when looking at *P. aeruginosa* with positive samples dropping from ~40%–32%, 26% and 23% after 3, 12 and 21 months of ETI therapy, respectively. They noted that changes in the detection were more apparent in the sputum samples vs. the throat cultures for both of these pathogens. Similar to what is seen in the US Cystic Fibrosis Foundation (CFF patient registry, this study noted the inverse age dependency of the likelihood of *P. aeruginosa* vs. *S. aureus* detection. Importantly, they also noted that for both pathogens, the majority of pwCF negative for or chronically colonized with either of these bacteria before ETI therapy remained in the same respective group after initiation of ETI [37].

A number of studies have been reported by Sheikh and colleagues on the impact of ETI on pwCF from Columbus, Ohio [31,38, 39]. Whilst these studies had different numbers of participants, each with different median ages, in all cases, it was observed that there were fewer pwCF with *P. aeruginosa*-positive or *S. aureus*-positive cultures after CFTR modulator therapy compared to before the initiation of therapy. However, these investigators also noted that more culture positivity was seen in patients who were able to expectorate sputum vs. those who could only provide throat cultures.

Investigators from Vertex and the CFF have reported interim results from the first 2 years of follow-up of their 5-year observation registry-based study [40]. The analysis focused on 9,311 pwCF, who had bacterial cultures available in each of the 5 years before and then following ETI treatment. They observed a reduction of all evaluated pathogens, including *P. aeruginosa*, *S. aureus*, * B. cepacia* complex and *Aspergillus*.

A more extensive comprehensive follow-up (over 3.5 years) on the pwCF in the PROMISE-micro study was recently reported [41]. In this case, the authors measured the prevalence of pathogens in sputum cultures and found that *P. aeruginosa* and * S. maltophilia* decreased after 1 month but not during the 3.5-year follow-up. On the other hand, *S. aureus* prevalence decreased at 2.5 years, but this was not seen at other time points. Using sputum culture for pathogen presence alone revealed that most pwCF were culture-negative for *P. aeruginosa* and *S. aureus*, but when both a more sensitive digital droplet PCR (ddPCR) and sputum culturing techniques were employed, very few patients appeared to have eradicated these pathogens. Interestingly, 50% of pwCF who had been initially *S. maltophilia*-positive eradicated this bacterium. Similar to what was found at 6 months [12], the proportions of pwCF that became culture negative did not appear to be increased during the 3.5 years of Trikafta [41]. This study noted that eradication was more likely if the pwCF had lower initial levels of lung colonization; most of the decrease in pathogen density occurred after 1 month. The authors noted that some pwCF can acquire new infections; however, whether these will become chronic infections and/or negatively impact lung health remains to be determined through continued monitoring of this cohort.

In addition to looking at the community composition, some studies have assessed the effect of CFTR modulators on pathogen physiology itself; these findings have been nicely summarized in a recent review [18]. One of the most interesting observations has been the direct antimicrobial effects of these modulators. Early work showed that IVA inhibited the growth of *S. aureus* with a less obvious impact on *P. aeruginosa* [42]. More recently, Cigana *et al*. followed up on these studies and showed similar results with ETI [43], although they also saw some synergy with typical antibiotics used for treating CF lung infections. These findings suggest that such interactions may be responsible for some of the changes in the microbial communities seen after modulator therapy.

### P. aeruginosa

*P. aeruginosa* is a Gram-negative pathogen associated with infections in the CF lung. These infections disproportionately lead to loss in lung function, lung damage and death compared to other pathogens [2,8, 14]. Since *P. aeruginosa* is such a detrimental pathogen to human health, reducing its prevalence or the morbidity of its infections is a significant priority for public health officials, researchers and the CF community. Studies have found that *P. aeruginosa* relative abundance decreases after the commencement of Trikafta [12]; however, infections did persist in most participants [10,12, 41]. In addition, a number of studies recognized a decrease in the number of participants who were infected with *P. aeruginosa* after they started Trikafta therapy [12,31, 33, 36,41, 44]. On the other hand, Martin *et al*. found no decrease in *P. aeruginosa* or total bacterial load in their cohort [23].

For *P. aeruginosa* specifically, Schnell *et al*. performed a prospective study of 69 pwCF (≥12 years old) from one CF centre in Germany after 24 weeks on ETI [45]. Colonization was performed by microbiological testing of sputum or deep throat swabs. In this patient cohort, most pwCF (57%) who were categorized as *P. aeruginosa*-positive before ETI treatment became chronically colonized. On the other hand, 36% of those patients positive before ETI were found to be *P. aeruginosa*-negative [37]. These findings are similar to those of others [12].

A study researching bacterial genomic changes found that at least one of the same lineages of *P. aeruginosa* remained in pwCF up to 21 months after starting therapy, suggesting that the same strains were maintained after treatment. Several genes were identified as commonly mutating in some of these lineages, which could be adaptive changes, but common phenotypical traits of chronic infections, like antibiotic resistance and O-antigen expression, remained the same in this experiment. Some of the specific mutations included genes for DNA mismatch repair, pathoadaptive proteins and secreted factors. This was a relatively small cohort (11 pwCF) from Australia, where a few started ETI after previously being on other modulator therapies [46], so the results and their implications are somewhat limited.

However, a similar result was found by Armbruster *et al*. when examining samples from 19 adults with CF before and after the initiation of ETI. Phylogenomic analysis of samples from a subset of this cohort revealed that six of seven individuals remained infected with the same *P. aeruginosa* genotype following ETI treatment. This group saw that these *P. aeruginosa* isolates were not genetically identical, and new variants were detected post-ETI suggesting evolution in the CFTR-corrected airway. They identified common mutations in genes involved in iron metabolism and acquisition, which suggests that changes in the small molecule environment could be facilitating the evolution of *P. aeruginosa* [47].

With these significant clinical improvements brought by Trikafta [10,12], the parallel changes in *P. aeruginosa* need to be considered to avoid future unintended consequences, like increased microbial virulence or better adaptation to the airway in the absence of the accumulation of sputum in the CF airway post-modulator therapy. This suggests that studying this highly prevalent, dangerous pathogen requires continued vigilance.

### S. aureus

*S. aureus* is another CF pathogen, which is less associated with morbidity than *P. aeruginosa* [2,8, 14], but is the predominant pathogen in young pwCF and the most common CF pathogen overall [1,9]. Thus, the presence of this Gram-positive microbe has significant implications for the health of the CF population. A number of groups have noted that the percentage of their cohorts of pwCF with *S. aureus* decreased [31,35,40], and similar results were obtained in the PROMISE-micro study. This latter study also noted that the absolute abundance of *S. aureus* also decreased and remained at depressed levels [12]. Contrasting this, Martin *et al*. found no decrease in abundance or bacterial load of *S. aureus*, similar to what they observed with *P. aeruginosa* [23]. On the other hand, Migliorisi *et al*. saw an increase in the prevalence of *S. aureus* in their cohort [33].

Some of the more important questions in the study of *S. aureus* in CF are why some isolates (either MSSA or MRSA) appear more pathogenic, causing chronic infections [48], and why infection prevalence seems to decrease with age; both of these issues are a significant barrier to its study. Perhaps because of this, there has been less emphasis on the genomic changes in *S. aureus* after starting Trikafta, but its prevalence in pwCF (particularly children) makes the study of it extremely important. Understanding how Trikafta guides the development of the early CF lung could be important for protecting the next generation of pwCF who are growing up in the world of Trikafta.

### Other bacteria and microbes

Fewer studies have been done on CF pathogens with lower prevalence like *S. maltophilia*, *Achromobacter* and the *B. cepacia* complex. In the PROMISE-micro study, the percentage of the cohort that had positive cultures for *Achromobacter* and *Burkholderia* stayed the same over 6 months on Trikafta. Relative and absolute abundance of *S. maltophilia* significantly decreased along with the common CF pathogens. Bower *et al*. saw the same decrease in prevalence [40]. For *Streptococcus*, *Prevotella*, *Veillonella* and other less common bacteria, the absolute abundances stayed the same [12]. Other broad microbiota studies that looked at the abundance of lesser-known pathogens did not provide information on whether there was a statistically significant difference in abundance after ETI [20,22, 23].

Trikafta therapy has had success in ameliorating NTM. Not only were patients less likely to contract NTM after commencing Trikafta compared to before, but after a year of therapy in 9 out of 15 patients, a negative NTM culture was also collected with no positives in the last year [49]. A case report [50] noted improved cavitary *Mycobacteroides abscessus* disease in a pwCF (F508del) even in the absence of an antimycobacterial regimen following Trikafta, suggesting that this treatment may restore lung function. *Aspergillus fumigatus* is a fungal pathogen associated with decreased lung function in CF. Trikafta was found to inhibit both growth and biofilms of *A. fumigatus* [51]. In one study, positive cultures before treatment became negative in four out of the six participants who started with *A. fumigatus* infections [22]. A decrease in *Aspergillus* prevalence was also noted by Bower *et al*. [40].

More recently utilizing ddPCR, the PROMISE-micro study noted that *A. fumigatus* was present in 28% of the pwCF at baseline, and this decreased to less than 5% after 1 month of treatment, with little additional decrease during the 30 months of the study. Among those infected with *A. fumigatus* at baseline, this became undetectable in 88% of the pwCF after treatment, suggesting that ETI may reduce the incidence of *A. fumigatus* in CF [52]. This eradication was much greater than that observed in pwCF with *P. aeruginosa* infection. Among those co-infected with *P. aeruginosa* at the same time, * A. fumigatus* became undetectable, but *P. aeruginosa* remained detectable, indicating the specificity of this response. Altogether, studies on the impact of Trikafta on these less common microbes have been relatively limited, and more are required to fully understand the dynamics of the respiratory microbial community after CFTR modulator therapy and the impact on CF health.

## Changes in the other microbiota

A few studies have looked at changes in the microbiota of other parts of the human body after starting Trikafta. Not only do these studies of other sites provide potential insight for and parallels to the pulmonary microbiome, but they also have intrinsic value for the study of each organ. Zemke *et al*. showed that after 9 months of Trikafta therapy, neither alpha nor beta diversity increased in the sinonasal microbiome; however, significant changes did occur with an increase in *Moraxella* (typical of healthy sinuses) abundance and a decrease in *Pseudomonas* and *Burkholderia* abundance. The relative abundance of *Staphylococcus*, the most abundant pathogen in the sinuses of the cohort, stayed the same [53].

Hilliam *et al*. [54] recently utilized sinus swabs and performed 16S and custom amplicon sequencing to characterize the sinus microbiota before and after ETI in 38 pwCF. They found that the Shannon diversity score was low and did not change after initiation of ETI. Whilst the abundance of *Pseudomonas* was reduced, this genus was still detectable, suggesting that it was not eradicated. *Staphylococcus* became the most prominent genus. The sinus microbiome correlated well with the major species of CF pathogens identified in the sputum (*Staphylococcus*, *Streptococcus* and *Pseudomonas* genera), but not necessarily all taxa within the samples. Critically, it was observed that *Staphylococcus* species increased or remained in high abundance following ETI. Whether this *Staphylococcus*-dominated community is specific to this patient cohort after ETI or for the sinuses and/or reflects similar changes in the sputum or lung environment in all pwCF will require additional studies comparing these types of samples. However, it is tempting to speculate that a sinus sample might be a good surrogate for predicting sputum microbiome composition [54]. The drop in abundance of *Pseudomonas* may suggest that a similar reaction to Trikafta is happening in the sinuses when compared to the lungs.

**Fig. 3.**
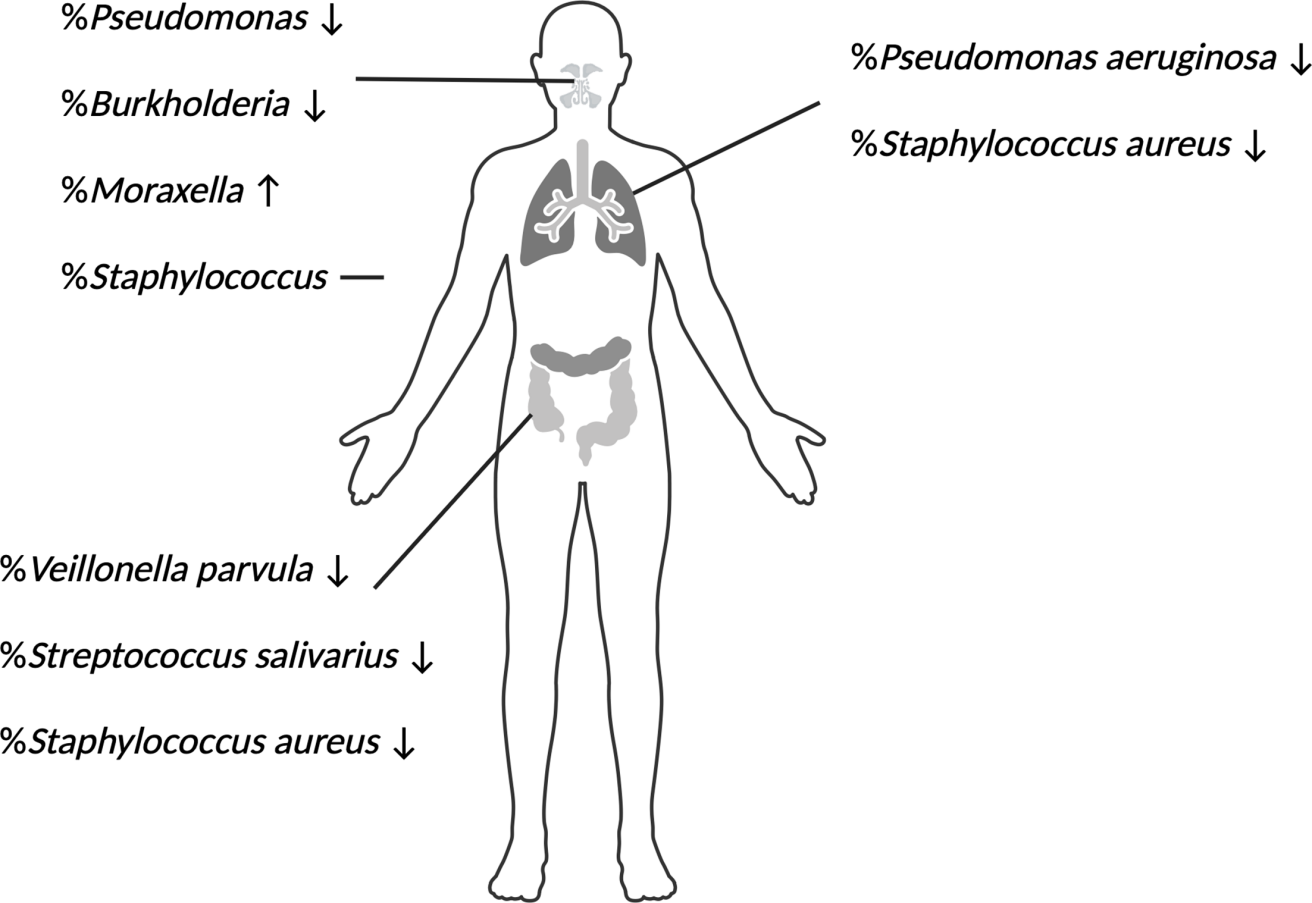
Effect of Trikafta on pathogen abundance in CF-associated infection sites. Sinuses, lungs and gut are pictured from top to bottom. A species or genus name listed with a down arrow indicates a decrease in relative abundance, a straight line indicates no change and an up arrow indicates an increase after ETI.

A study of pwCF-associated liver disease analysed changes in the faecal microbiome. They found high initial variation between individuals and no significant change in alpha diversity and richness 1 month or 6 months after beginning Trikafta therapy. *S. aureus*, *Streptococcus salivarius* and *Veillonella parvula* relative abundances decreased [55]. Marsh *et al*. [56] analysed faecal samples from 20 pwCF before and then three times following the initiation of Trikafta treatment. Whilst the gut microbiome remained significantly different from that of healthy matched controls, there was a trend from the pwCF microbiome towards the healthy microbiome following Trikafta (Fig. 3). A decrease in the relative abundance of pathogens seems to be associated with multiple microbiomes in the body, which can give us more confidence that the abundance of pathogens in the pulmonary microbiota is decreasing. Large and comprehensive studies looking at multiple microbiomes simultaneously could provide relationships between the changes in all these diverse environments.

## Conclusions, challenges and future directions

The initiation of CFTR modulator therapy has drastically changed the health of most pwCF, leading to improvements in clinical outcomes including increased ppFEV1, nutritional status and a reduction in pulmonary exacerbations. However, chronic pulmonary infections persist in some pwCF [57], and respiratory infections remain the major cause of morbidity and mortality. Despite this, the overall positive impact of CFTR modulators cannot be overstated. With that said, the impact of these therapies on the respiratory microbiota must be considered to change the course of this disease. The decreased prevalence of CF pathogens and the associated changes in the composition of the microbiota provide significant optimism for the future. Increasing diversity in the pulmonary microbiota, to one more similar to that of healthy individuals, likely contributes to the increased lung function and improved quality of life associated with Trikafta [10,12]. The decrease in CF respiratory pathogen abundance, likely mediated by less available metabolites, an inhibited immune response and direct inhibition by the modulators, creates a healthier microbiota. How the microbiota of children who never developed chronic infections emerges remains to be seen. It is possible that many of these children may not be subjected to high burdens of pathogenic bacteria in their lungs, but the future prevalence of existing or new strains and species is a significant possibility that requires careful attentiveness from the scientific community.

In general, the ubiquitous CF pathogens, *P. aeruginosa* and *S. aureus*, have been shown to be decreased following Trikafta, but more sensitive measurements do not indicate complete eradication. Less studied but still important pathogens like * S. maltophilia*, NTM and *A. fumigatus* also have evidence of being reduced by Trikafta therapy. However, inherent in all these studies is that there is day-to-day variation in pathogen density in individual patients’ sputum samples [58] as well as the decreased availability of sputum that is able to be cultured as a read-out of the inhabitants of the lung. Swabs have been an effective replacement in several studies [24,25], but the increased burden of swabs, questions of accuracy and uncertain correlation with sputum samples before ETI are significant challenges. We recommend a comprehensive study of several sampling methods with participants pre- and post-ETI to address this problem. Compounding the sampling issues is that the antibiotic resistance/sensitivity profile as determined by the clinical microbiology laboratory does not always predict whether particular antibiotics will be effective at clearing lung infections in a pwCF.

The reach of Trikafta therapy extends past the lung, with pathogen abundance in the sinuses and stool also seeming to decrease. Trikafta is the perfect example of a medication that tackles the underlying problem rather than the symptoms. In the setting of the antibiotic crisis, more therapies like Trikafta are needed to protect our vulnerable populations; however, when developing these therapies, their secondary and long-term effects need to be heavily considered. It is still unclear how Trikafta will change the microbiota on increasingly long time scales, so continuous research and stewardship are needed to make sure the impact remains positive.

Challenges to this approach remain, as some pwCF are not eligible to receive CFTR modulator therapy. Unfortunately, minority populations are more seriously affected, as they often are carrying rare or poorly studied CFTR mutations. This, combined with the fact that these rare CFTR variants are not included in newborn screening panels, leads to significant racial and ethnic disparities in the treatment for this disease [59]. However, it is exciting that new CFTR modulators continue to be developed [9].

The increase in life expectancy does pose new challenges for healthcare professionals in treating the CF population. For example, weight gain as a result of CFTR modulator therapy has been linked to associated comorbidities that affect the general population, like increased heart disease [60]. Significant changes are also occurring in the personal lives of pwCF. For instance, CF has typically caused significant infertility [1], but ETI therapy, treating the fundamental cause along with greater ability for an active lifestyle [11], means that family planning will likely become much more common. With that said, the impact of the CFTR modulators on lung physiology and the concomitant decreases in microbial infections have significantly changed a pwCF’s perception of what managing and living with CF means.

## Abbreviations

AA: amino acid
CF: cystic fibrosis
CFF: Cystic Fibrosis Foundation
CFTR: cystic fibrosis transmembrane conductance regulator
ddPCR: digital droplet PCR
ETI: elexacaftor, tezacaftor and ivacaftor
EXA: elexacaftor
IVA: ivacaftor
MRSA: methicillin-resistant *Staphylococcus aureus*
MSSA: methicillin-sensitive *Staphylococcus aureus*
NTM: nontuberculous mycobacterium
ppFEV1: percent predicted forced expiratory volume
pwCF: people with CF
TEZ: tezacaftor
